# The Effect of Sex Differences on Endothelial Function and Circulating Endothelial Progenitor Cells in Hypertriglyceridemia

**DOI:** 10.1155/2020/2132918

**Published:** 2020-09-21

**Authors:** Zi Ren, Jiayi Guo, Xingxing Xiao, Jiana Huang, Manchao Li, Ruibin Cai, Haitao Zeng

**Affiliations:** ^1^Center for Reproductive Medicine, Sixth Affiliated Hospital, Sun Yat-sen University, Guangzhou 510655, China; ^2^Division of Emergency Medicine, Department of Emergency Intensive Care Unit, The First Affiliated Hospital, Sun Yat-Sen University, Guangzhou 510080, China

## Abstract

**Background:**

Men have a higher risk and earlier onset of cardiovascular diseases compared with premenopausal women. Hypertriglyceridemia is an independent risk factor for the occurrence of ischemic heart disease. Endothelial dysfunction is related to the development of ischemic heart disease. Whether sex differences will affect the circulating endothelial progenitor cells (EPCs) and endothelial function in hypertriglyceridemia patients or not is not clear.

**Methods:**

Forty premenopausal women and forty age- and body mass index (BMI)-matched men without cardiovascular and metabolic disease were recruited and then divided into four groups: normotriglyceridemic women (women with serum triglycerides level <150 mg/dl), hypertriglyceridemic women (women with serum triglycerides level ≥150 mg/dl), normotriglyceridemic men (men with serum triglycerides level <150 mg/dl), and hypertriglyceridemic men (men with serum triglycerides level ≥150 mg/dl). Peripheral blood was obtained and evaluated. Flow-mediated dilatation (FMD), the number and activity of circulating EPCs, and the levels of nitric oxide (NO), vascular endothelial growth factor (VEGF), and granulocyte-macrophage colony-stimulating factor (GM-CSF) in plasma and culture medium were measured.

**Results:**

The number and activity of circulating EPCs, as well as the level of NO in plasma or culture medium, were remarkably increased in premenopausal females compared with those in males both in the hypertriglyceridemic group and the normotriglyceridemic group. The EPC counts and activity, as well as the production of NO, were restored in hypertriglyceridemic premenopausal women compared with those in normal women. However, in hypertriglyceridemic men, the EPC counts and activity, as well as levels of NO, were significantly reduced. The values of VEGF and GM-CSF were without statistical change.

**Conclusions:**

The present study firstly demonstrated that there were sex differences in the number and activity of circulating EPCs in hyperglyceridemia patients. Hypertriglyceridemic premenopausal women displayed restored endothelial functions, with elevated NO production, probably mediated by estradiol. We provided a new insight to explore the clinical biomarkers and therapeutic strategies for hypertriglyceridemia-related vascular damage.

## 1. Introduction

It is well known that atherosclerosis is a progressive inflammatory disease characterized by plaque consisting of cholesterol, fat, calcium, and other substances deposition in the arterial wall. Epidemiological data have suggested that there are remarkably sex differences in the onset of cardiovascular disease (CVD), with atherosclerosis occurring approximately ten years later and myocardial infarction about 20 years later in premenopausal women than in man [1]. However, the risk of CVD rises about ten times in postmenopause women, while only increases 4.6 times in age-matched men [2]. The mechanisms of sex differences in the occurrence of CVD are not fully elucidated. Researches have indicated that estrogen [3] and androgen [4] may play a crucial role in the development of CVD.

Hypertriglyceridemia is commonly defined as fasting triglyceride serum level >150 mg/dl (>1.7 mmol/l) [5]. There is growing evidence suggesting that hypertriglyceridemia is an independent risk factor for ischemic cardiovascular disease [6–11], even slightly elevation of triglycerides was associated with a higher cardiovascular risk [8] and mortality rate [9]. In the general population, compared with the individuals with nonfasting triglycerides of 70 mg/dL (0.8 mmol/L), individuals with levels of 580 mg/dL (6.6 mmol/L) have sharply increased risks of myocardial infarction for 5.1-fold, ischemic heart disease, and ischemic stroke for 3.2-fold, respectively, and all-cause mortality for 2.2-fold [11]. Lipid-lowering treatment with statins was proved to exert a positive effect on patients with atherosclerosis through restoring the endothelial function and increased circulating endothelial progenitor cells (EPCs) [12, 13]. However, some researches have indicated patients with atherosclerosis may not get enough protection by statins alone [14], especially in patients with systemic inflammation [15]. Besides, statins may have some side effects such as statin-associated symptoms (SAS), including muscle or central nervous system symptoms and diabetes [16].

Vascular endothelium cell, nature's blood container, is the dynamic regulator of hemostasis and thrombosis with the function of recruitment and activation of platelet and coagulation cascade [17]. Therefore, vascular endothelium dysfunction was proven to be a significant risk factor for vascular diseases, such as atherosclerosis [18] and insulin resistance [19, 20]. Asahara et al. have firstly described that EPCs isolated from peripheral blood play a crucial role in angiogenesis [21]. Early EPCs, derivatives of CD14+ monocytic cell lineage [22], promote angiogenesis by secreting inflammatory cytokines and paracrine angiogenic factors [23]. While late EPCs, originating from CD34+ hematopoietic stem cells [24, 25], are associated with endothelial tubulogenesis and neovascularization via enhanced expression of proliferation and angiogenesis genes [23]. EPCs promote angiogenesis or vascular repair through activation of resident endothelial cells and recruitment of endogenous monocytes/macrophages to sites of ischemia, which were mediated by paracrine factors [26] such as vascular endothelial growth factor (VEGF) [27–29], granulocyte-macrophage colony-stimulating factor (GM-CSF) [29–32], and nitric oxide (NO) [32–34]. Dyslipidemia, hyperglycemia, insulin resistance, and induced EPC dysfunction via disrupting the SDF-1/CXCR-4 and NO pathways and the p53/SIRT1/p66Shc axis are critical for mobilization, migration, homing, and vasculogenic properties [35]. Researches also have indicated that EPC therapy is a safe and efficient way to delay the progression of atherosclerosis [36] and improve the heart function [37] for patients with coronary heart disease.

Our previous study has demonstrated that premenopausal women in prehypertension status present an increased circulating EPC number and elevated NO level, which may associate with the vascular protection effect of premenopausal women [38]. In this study, we will further investigate the sex differences of the endothelial function and circulating EPCs in patients of hypertriglyceridemia, and the probable underlying mechanisms.

## 2. Materials and Methods

The materials and methods section should contain sufficient detail so that all procedures can be repeated. It may be divided into headed subsections if several methods are described.

### 2.1. Characteristics of Subjects

Forty premenopausal women and forty age- and BMI-matched men without cardiovascular and metabolic disease were recruited. Based on the Adult Treatment Panel III (ATP-III) guidelines [5], according to the triglyceride level and sex, we divided the subjects into four groups: normotriglyceridemic women (women with serum triglycerides level <150 mg/dl), high triglyceridemic (HTG) women (women with serum triglycerides level ≥150 mg/dl), normotriglyceridemic men (men with serum triglycerides level <150 mg/dl), HTG men (men with serum triglycerides level ≥150 mg/dl). Patients with diabetes, tumor or cancer, and infection or inflammatory disease were excluded. Besides, we also excluded the smokers, alcohol abusers, pregnant, women undergoing breastfeeding, or patients with a history of hysterectomy, oophorectomy, or irregular menstrual cycles. All the subjects were given informed consent, and the experimental protocols were approved by the ethics committee of our hospital. The characteristics of the subjects are listed in Table 1.

### 2.2. Blood Samples

The peripheral blood was obtained from the patients in the early morning after overnight fasting. Caffeinated beverage or alcohol was forbidden for at least 12 hours before blood draw. None of the patients were taking any medicines such as statins, antiplatelet, or anti-inflammatory that may have an impact on the parameters of EPCs. The following items were detected and evaluated: AST (aspartate aminotransferase), ALT (alanine aminotransferase), BUN (blood urea nitrogen), Cr (creatinine), LDL (low-density lipoprotein), TC (total cholesterol), HDL (high-density lipoprotein), TG (triglyceride), FPG (fasting plasma glucose), estradiol, and so on, which are presented in Table 1.

### 2.3. Evaluation of the EPC Number and Activity

The number of circulating EPCs was evaluated by flow cytometry analysis and cell culture assay, and EPC activity was measured by EPC migration and proliferation assay, which were demonstrated in our previous studies [38, 39].

### 2.4. Measurement of the Plasma Levels of NO, VEGF, and GM-CSF and Secretion by EPCs

The plasma levels of NO, VEGF, and GM-CSF and secretion by cultured EPCs were evaluated by methods as we described previously [38, 39].

### 2.5. Measurement of FMD

Flow-mediated dilation (FMD) of the brachial artery was used to evaluate the endothelial function. The detailed method was demonstrated in our previous studies [40, 41].

### 2.6. Statistical Analysis

SPSS Version 26.0 statistical software (SPSS Inc., Chicago, Illinois) was used for data analysis. Results were expressed as mean value ± standard deviation. Two-factor ANOVA was used for comparisons between the four groups (sex and the status of normal triglyceridemia or hypertriglyceridemia). When there was a significant F value, a post hoc test was then performed with the Newman–Keuls method to identify significant differences among mean values. Univariate correlations were analyzed by Pearson's coefficient (*r*). *P* < 0.05 was considered as statistically significant.

## 3. Results and Discussion

### 3.1. Baseline Clinical Characteristics

As we have shown in Table 1, there were no significant differences in age, BMI, and serum TG, TC, LDL, and HDL among the four groups (*P* > 0.05). The serum TG levels of both premenopausal HTG women (*P* < 0.05) and HTG men (*P* < 0.05) were remarkably increased than those in the normotriglyceridemic groups. The serum level of estradiol and the value of FMD were higher in the female groups than in the male groups (both normotriglyceridemic and HCG groups, *P* < 0.05 and *P* < 0.05, respectively). Moreover, FMD was obviously lower in the HMG men compared with that in the normotriglyceridemic men (*P* < 0.05). However, the value of FMD in normotriglyceridemic women and HMG women did not have remarkable differences (*P* > 0.05).

### 3.2. EPC Number and Activity in the Four Groups

**Figure 1:**
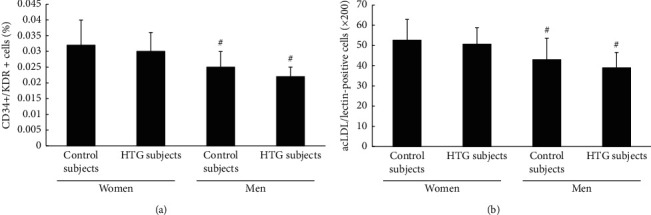
(a) fluorescence-activated cell sorting analysis. (b) Phase-contrast fluorescent microscope. There was no significant difference in the level of circulating EPCs between the normotriglyceridemic and the hypertriglyceridemic women. In both the normotriglyceridemic and hypertriglyceridemic patients, the number of EPCs in women groups was statically higher than that in the men groups. Data were presented as mean ans standard deviation. #vs premenopausal women.

The number of EPCs evaluated by FACS analysis and cell culture assay of four groups is shown in Figure 1. The number of EPCs in the normotriglyceridemic groups was close to that in the hypertriglyceridemic groups of the same sex, both men and women (*P* > 0.05 and *P* > 0.05, respectively) (Figures 1(a) and 1(b)). However, the number of EPCs in men, both normotriglyceridemic and hypertriglyceridemic groups, was drastically lower than that in women (*P* < 0.05) (Figures 1(a) and 1(b)).

**Figure 2:**
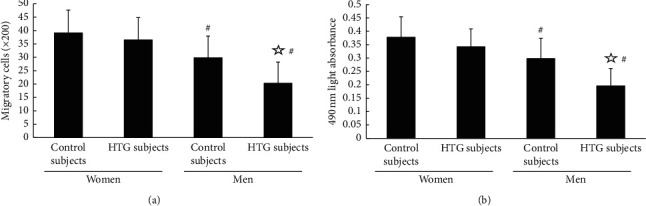
(a) The migratory and (b) the proliferative activities of EPCs were statically elevated in the female groups (both normotriglyceridemic and hypertriglyceridemic) than those in the male groups. The migratory and the proliferative activities of EPCs were lower in the hypertriglyceridemic men group than those in the normotriglyceridemic men group. However, the migratory and the proliferative activities of EPCs in normotriglyceridemic premenopausal women were similar to those in hypertriglyceridemic premenopausal women. Data were presented as mean and standard deviation. ☆vs. normotriglyceridemic. #vs premenopausal women.

The migratory and the proliferative activities of EPCs were significantly higher in both normotriglyceridemic and hypertriglyceridemic premenopausal female groups than those in the male groups (*P* < 0.05 and *P* < 0.05, respectively) (Figures 2(a) and 2(b)). Besides, in the male groups, the migratory and the proliferative activities of EPCs were statistically decreased in the hypertriglyceridemic men compared with those in the normotriglyceridemic men (*P* < 0.05) (Figures 2(a) and 2(b)). However, the activity of EPCs in normotriglyceridemic premenopausal women was not remarkably different from that in hypertriglyceridemic premenopausal women (*P* > 0.05) (Figures 2(a) and 2(b)).

### 3.3. Levels of NO, VEGF, and GM-CSF in Plasma or Culture Media in the Four Groups

**Figure 3:**
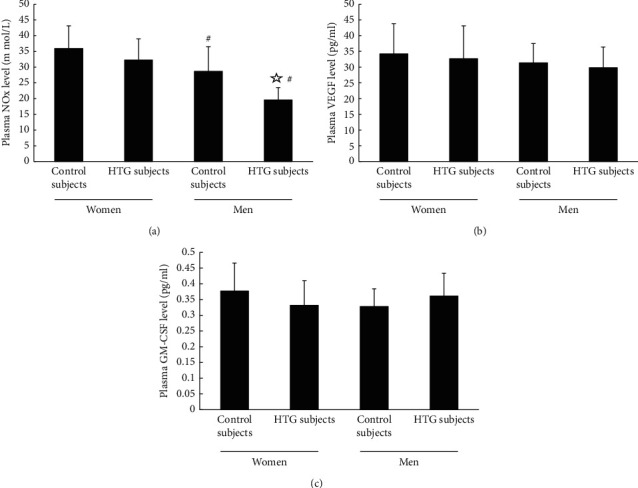
The plasma levels of NO, VEGF, and GM-CSF. (a) The plasma levels of NO in normotriglyceridemic and hypertriglyceridemic men were statically lower than those in the female groups (*P* < 0.05). In the male groups, the level of NO was decreased in hypertriglyceridemic subjects than that in normotriglyceridemic subjects (*P* < 0.05). The plasma levels of NO between normotriglyceridemic and hypertriglyceridemic premenopausal female groups displayed no significant difference (*P* > 0.05). (b) and (c) The plasma level of VEGF and GM-CSF had no statistical difference between the four groups.

**Figure 4:**
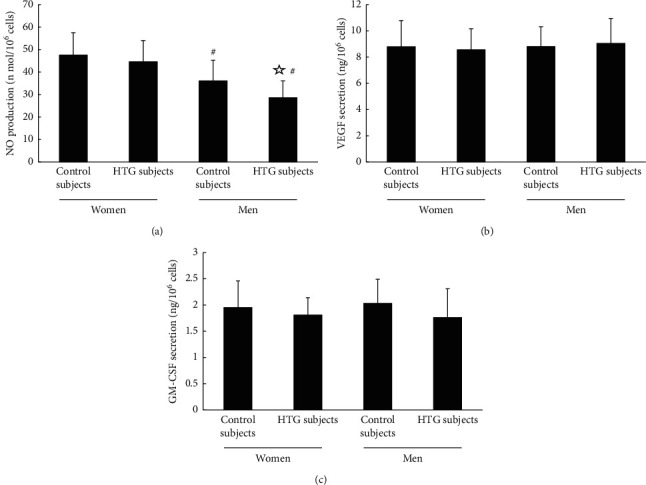
The levels of NO, VEGF, and GM-CSF secreted by cultured EPCs. (a) The level of NO produced by cultured EPCs of the normotriglyceridemic and hypertriglyceridemic men groups was remarkably lower than that of the premenopausal women groups. Besides, the secretion of NO from hypertriglyceridemic men was statically elevated compared with that from normotriglyceridemic men. Nevertheless, the level of NO in the EPCs cultural media of the hypertriglyceridemic premenopausal women was not significantly different from that of the normotriglyceridemic premenopausal women). (b) and (c) There was no obvious difference in VEGF or GM-CSF secretion between four groups.

The level of NO from plasma or culture media of the normotriglyceridemic and hypertriglyceridemic men groups was remarkably lower than that of the premenopausal women groups (*P* < 0.05 and *P* < 0.05, respectively) (Figures 3(a) and 4(a)). However, the differences in plasma level of NO or the level of NO secreted by cultured EPCs between the normotriglyceridemic and hypertriglyceridemic premenopausal female groups did not reach statistical significance (*P* > 0.05 and *P* > 0.05, respectively) (Figures 3(a) and 4(a)). In addition, the level of NO in plasma or cultured media of the hypertriglyceridemic men was statistically elevated compared with that from the normotriglyceridemic men (*P* < 0.05 and *P* < 0.05, respectively) (Figures 3(a) and 4(a)). Nevertheless, there were no drastic differences in VEGF or GM-CSF plasma level or secretion between four groups (Figures 3(b), 3(c), 4(b), and 4(c)).

### 3.4. Correlation between FMD and EPCs or NO Level

**Figure 5:**
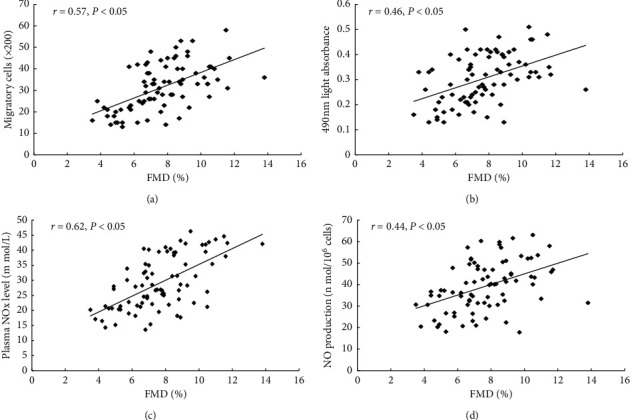
The correlation between FMD and EPCs or NO level. (a) and (b) There was a positive relevance between FMD and the proliferative or migratory activity of circulating ECPs. Besides, (c) and (d) FMD was positively correlated with the plasma NO level or NO level detected from cultured media.

FMD, which reflected the endothelial function, was positively relevant with the migratory (*r* = 0.57, *P* < 0.05) and the proliferative (*r* = 0.46, *P* < 0.05) activity of circulating ECPs (Figures 5(a) and 5(b)). Moreover, there was a positive correlation between FMD and the plasma NO level (*r* = 0.62, *P* < 0.05) or level of NO secreted by cultured EPCs (*r* = 0.44, *P* < 0.05) (Figures 5(c) and 5(d)).

## 4. Discussion

Our research firstly demonstrated sex effects on the endothelial function and circulating endothelial progenitor cells in hypertriglyceridemic patients, which may be probably attributed to the disturbance of NO production. We have evaluated that the number and activity of EPCs were preserved in premenopausal women compared with those in the age-matched men in hypertriglyceridemic status. Besides, the plasma level of NO or NO level secreted by EPCs in the culture media was higher in the premenopausal hypertriglyceridemic women than in men. Moreover, there was a positive correlation between EPCs activity or the NO level and endothelial functions evaluated by FMD.

It is well documented that there are sex differences in the occurrence of ischemic heart disease. Generally, males tend to have earlier onset [1] of occlusive coronary artery disease (CAD) [42] with more severe infarction foci and poor recovery [43–45] than females. EPCs are essential for the intrinsic repair and regeneration process of the injured myocardium [46]. Ischemic heart disease was proved to be associated with depletion of circulating EPCs [47, 48] and higher local density of EPCs [48]. Coincidently, in comparison with men or postmenopausal women, premenopausal women exhibit a higher level of circulating EPCs with better colony-forming capacity and migratory activity [49–52]. This phenomenon was consistent with that of the previous studies of the sex differences in the occurrence of ischemic heart disease, which implied that the better cardiovascular repaired and protective capacity in premenopausal women might partly attribute to the enhanced number and activity of EPCs. Hypertriglyceridemia is proved to be an independent risk factor of cardiovascular disease [6–11]. However, there were no scientific data revealing the relationship between sex differences and hypertriglyceridemia and EPCs. Hence, we postulated that the sex differences in circulating EPCs might also exist in hypertriglyceridemia.

We have studied the number and activity differences in circulating EPCs among four groups. Generally, the number of circulating EPCs in male groups (both the hypertriglyceridemia and the control) was higher than that in the age-matched premenopausal female groups, while the activity of EPCs was depleted compared with that of female groups, which were consistent with the previous studies [49–52]. Furthermore, we noticed that, in the male groups, the number and activity of EPCs are drastically reduced in patients with hypertriglyceridemia, which suggested the depletion of the number and activity of EPCs in hypertriglyceridemia may at least partly explain the higher risk of ischemic heart disease in hypertriglyceridemic male patients. Interestingly, we found that the number and activity of circulating EPCs in hypertriglyceridemic premenopausal women was nearly identical to that in normotriglyceridemic premenopausal women. The present results suggested the restoration of endogenous vascular endothelial repair capacity in premenopausal women, even in hypertriglyceridemia status. The reduction of number and activity of EPCs only existed in hypertriglyceridemic males rather than in the hypertriglyceridemic premenopausal female, indicating that a higher tendency of ischemic heart disease in hypertriglyceridemic men than in hypertriglyceridemic premenopausal women may be due to the sex differences in endogenous endothelial repair capacity.

It is well known that EPCs promoting angiogenesis or vascular repair is mediated by paracrine molecules such as VEGF [27–29], GM-CSF [29–32], and NO [32–34]. Previous studies elucidated that elevation of triglycerides was inversely correlated with the level of NO [53–55]. Therefore, we hypothesized that the sex differences in EPCs in patients with hypertriglyceridemia might be related to the alteration of the production of VEGF, GM-CSF, or NO. We detected the plasma level of NO, VEGF, and GM-CSF in the four groups. Similar to the sex differences in the changes of circulating EPCs in hypertriglyceridemia status, we found that the NO plasma level was lower in male groups than in premenopausal female groups, which was consistent with those of the previous studies in healthy subjects [56]. Besides, the plasma NO level was restored in premenopausal women in hypertriglyceridemia status compared with that in women in the control group. In our previous research, we have elucidated the positive correlation between plasma NO level and the number or activity of circulating EPCs [38]. Therefore, it suggested that the restoration of number and activity of circulating EPCs in hypertriglyceridemic premenopausal women may be attributed to the stability of exogenous NO production. Nevertheless, we did not observe a significant change in the plasma level of VEGF or GM-CSF among four groups, implicating that VEGF and GM-CSF may not be the key factors contributing to the sex differences in hypertriglyceridemia.

NO was generated by endothelial nitric oxide synthase (eNOS), which acts as a key regulator for the homeostasis of endothelial function [57]. Therefore, we further investigated the sex differences in NO production by EPCs. And, we found that the level of NO secreted by EPCs was similar to the change of the NO plasma level. As we have mentioned before, NO secretion by EPCs is significantly correlated with the number and activity of circulating EPCs [38]. Our result suggested that enhanced endogenous NO production would probably help to preserve the number and activity of circulating EPCs in hypertriglyceridemic women. In addition, there was no remarkable difference in the production of VEGF and GM-CSF, which further suggested that VEGF and GM-CSF did not participate in the sex differences in the EPCs of hypertriglyceridemia.

FMD is an indicator of endothelial function. We further investigated the relationship between FMD and EPCs or NO. The result revealed that the activity of EPCs and NO exogenous or endogenous production was positively correlated with the endothelial function.

In general, we discovered the conservation of endothelial function with EPC counts and activity in hypertriglyceridemic premenopausal women, which may be attributed to the enhanced production of NO. It is well documented that NO plays an essential role in the maintenance of vascular homeostasis, including regulation of vascular dilator tone, modulation of local cell growth, and protection of vessels [58]. Endothelial cells, as well as vascular smooth muscle cells, are crucial targets of estradiol [59]. Furthermore, estradiol activates early and late endothelial eNOS via binding the estrogen receptors through nongenomic and genomic pathways [60]. Undoubtedly, the remarkable difference between premenopausal women and age-matched men is the estradiol level. Hence, we inferred that estradiol augmented NO production and thereby increased the number and activity of circulating EPCs in hypertriglyceridemia.

The present study demonstrated a new insight into the evaluation and therapeutic targets of hypertriglyceridemia-related endothelium injury. Firstly, we showed the sex differences in circulating EPC counts and activity, indicating that EPCs and FMD could act as an essential clinical biomarker to detect hypertriglyceridemia-related vascular injury. Secondly, the correlation of higher cardiovascular risk with the reduction of EPC counts and activity in men compared with that in premenopausal women reminded us that enhancement of circulating EPC counts and activity is a potential therapeutic target to reverse the hypertriglyceridemia-related endothelial damage. Furthermore, our study also revealed that enhanced NO production was essential for the improvement of EPC number and activity. Hence, we advocated that patients with hypertriglyceridemia could adopt some strategies, such as exercises [39, 61], to stimulate NO production and thereby improve the endothelial repair capacity.

## 5. Conclusions

The present study raised a new view that there were sex differences in the number and activity of circulating EPCs in hyperglyceridemia patients. Hypertriglyceridemic premenopausal women displayed restored endothelial functions, which was associated with the enhanced NO production, probably mediated by estradiol. We provided a new insight to explore the clinical biomarkers and therapeutic strategies for hypertriglyceridemia-related vascular damage.

## Figures and Tables

**Table 1:** Clinical and biochemical characteristics.

Characteristics	Normotriglyceridemic women (*n* = 20)	HTG women (*n* = 20)	Normotriglyceridemic men (*n* = 20)	HTG men (*n* = 20)
Age (years)	44.8 ± 3.4	45.3 ± 3.28	46.6 ± 4.4	46.2 ± 3.6
Height (cm)	162.7 ± 5.4	160.6 ± 6.7	168.1 ± 6.0^#^	169.2 ± 6.2^#^
Weight (kg)	68.6 ± 6.5	69.1 ± 6.0	75.0 ± 5.3^#^	75.3 ± 4.3^#^
BMI (kg/cm^2^)	25.9 ± 2.3	26.8 ± 2.2	26.2 ± 2.2	26.7 ± 1.6
Systolic blood pressure (mmHg)	123.2 ± 9.0	125.2 ± 3.4	124.3 ± 8.7	127.0 ± 4.4^☆^
Diastolic blood pressure (mmHg)	75.3 ± 7.2	78.0 ± 5.9	76.1 ± 7.5	78.3 ± 5.1^☆^
Heart rate (beats/min)	78.8 ± 9.3	81.3 ± 8.3	77.5 ± 7.7	78.3 ± 8.8
AST (mmol/L)	28.1 ± 6.2	26.7 ± 5.1	25.8 ± 6.3	28.8 ± 3.1
ALT (mmol/L)	23.3 ± 6.9	22.4 ± 5.5	21.4 ± 5.1	24.3 ± 5.7
BUN (mmol/L)	4.6 ± 0.8	5.0 ± 1.1	4.9 ± 0.7	5.1 ± 0.8
Cr (mmol/L)	60.4 ± 11.7	64.6 ± 13.6	61.3 ± 12.0	64.7 ± 12.1
LDL (mmol/L)	2.81 ± 0.45	2.69 ± 0.45	2.73 ± 0.42	2.57 ± 0.40
TC (mmol/L)	4.70 ± 0.50	4.50 ± 0.56	4.51 ± 0.62	4.34 ± 0.63
HDL (mmol/L)	1.31 ± 0.23	0.97 ± 0.15^☆^	1.34 ± 0.15	0.93 ± 0.14^☆^
TG (mmol/L)	1.53 ± 0.17	3.42 ± 0.73^☆^	1.48 ± 0.16	3.66 ± 0.75^☆^
FPG (mmol/L)	4.66 ± 0.84	4.47 ± 0.50	4.44 ± 0.45	4.83 ± 0.45
Estradiol (pmol/L)	224.48 ± 33.4	212.3 ± 35.7	104.2 ± 19.9^#^	110.92 ± 16.6^#^
FMD (%)	8.97 ± 1.99	8.22 ± 1.56	7.60 ± 1.66^#^	5.85 ± 1.77^#☆^

BMI, body mass index; LDL, low-density lipoprotein; TC, total cholesterol; HDL, high-density lipoprotein; TG, triglyceride; FPG, fasting plasma glucose; hrCRP, hypersensitive C-reactive protein; FMD, flow-mediated brachial artery dilatation; HTG, hypertriglyceridemic. Notes: data are given as mean ± SD. ☆vs normotriglyceridemic subjects; # vs. premenopausal women.

## Data Availability

The datasets used in the current study can be made available from the corresponding author on reasonable request.
